# Examining the Effects of Theory of Mind and Social Skills Training on Social Competence in Adolescents with Autism

**DOI:** 10.3390/bs13100860

**Published:** 2023-10-20

**Authors:** Weina Ma, Jieyu Mao, Yu Xie, Simeng Li, Mian Wang

**Affiliations:** 1 Jing Hengyi School of Education, Hangzhou Normal University, Hangzhou 311121, China; mwn505@hznu.edu.cn; 2 Zhejiang Philosophy and Social Science Laboratory for Research in Early Development and Childcare, Hangzhou Normal University, Hangzhou 311121, China; 3 Yang Ling Zi School, Hangzhou 311121, China; 4 Gevirtz Graduate School of Education, University of California, Santa Barbara, CA 93106, USA

**Keywords:** autism, theory of mind, social skills, social competence, multiple baseline designs

## Abstract

Individuals with autism spectrum disorders (ASD) have impairment in interpreting emotional communication and the mental states of others, which limits their social competence. Mounting evidence has suggested that theory of mind (ToM) is a vital strategy to enhance social communication and interaction skills of children with ASD. However, very little research has looked at how ToM and social skills training affect social competence in adolescents with autism. This study examined the effectiveness of an intervention program, ToM-SS, which integrated the ToM and social skills training to improve the social competence of three adolescents with autism. A multiple baseline across behaviors design was adopted to evaluate the participants’ learning outcomes and demonstrated a functional relationship between intervention and skill mastery. Results show that the intervention produced substantial improvements in students’ acquisition of ToM (e.g., seeing leads to knowing and identifying desire-based and context-based emotions) and targeted social skills (e.g., praising others, expressing emotion and seeking help). Feedback and comments from teachers and parents also indicate good social validity of the intervention program.

## 1. Introduction

Autism spectrum disorders (ASD) are neurodevelopmental disorders and have impairments in social communication, interpreting emotional communication and the mental states of others [1]. Individuals with ASD usually have difficulties in understanding social communication and behaviors, resulting social retreat and social anxiety [2]. The prevalence of ASD has been rising every year [3]. Therefore, it is very important to improve the social competence of individuals with ASD for their mental health and daily life.

A number of studies have demonstrated that the social skills interventions can improve social competence and quality of life for individuals with ASD [4]. These studies can be divided into three categories. First, using virtual reality to intervene in the social competence of individuals with ASD. Wang and Xing [5] examined youth with ASD learning social ability in the context of the game-based activities in a 3D virtual world. They found that the gamed-based learning activities improved social performance of youth with ASD. Similarly, Kourtesis et al. [6] suggested that immersive virtual reality appears to be an appropriate service, which can be used in social skill training in individuals with ASD. Moreover, virtual reality has been used in improving emotion recognition and speech in children with ASD [7]. Compared to the traditional emotion recognition, children with ASD in virtual reality training group spent less time to improve the performance of their emotion recognition [8]. Augmented Reality also have a positive effect on increasing the motivation in children with ASD [9]. Second, using robot to intervene in the social competence of individuals with ASD. Holeva et al. [10] suggested that a robot-assisted psychological intervention helped improve psychosocial skills in children with ASD. Robot-assisted therapy has also been found to have the potential to improve social interaction, communication and emotion regulation in children with ASD [11,12]. Third, using video modeling to intervene in the social competence of individuals with ASD. Whittenburg et al. [13] investigated the effects of behavioral skills training with video modeling on workplace conversational skills of four students with autism. They demonstrated that video modeling can be incorporated with behavioral skills training and increase in conversation skills in students with autism. A case study conducted by Rega et al. [14] used video modeling to successfully improve the development of emotional skills in children with ASD.

In addition, autism has been attributed to a lack of theory of mind (ToM), a skill that develops around six years of age in typically developing children [15]. Unlike their peers, children with ASD struggle in social-emotional reciprocity and are unable to understand that others have beliefs, desires and intentions that may differ from their own, which limits their competence in forming and maintaining social relationships and collectively hinders daily functioning [16,17]. Theory of Mind was first introduced by Premack and Woodruff. It refers to an individual’s ability to postulate or make assumptions across a full range of mental states—intentions, beliefs, needs and desires—and then interpret others based on these understandings [17]. ToM is a prerequisite skill for establishing many types of social relationships, lacking this skill contributes to symptoms of impaired socialization, communication and restricted and repetitive behaviors (RRBs) [18,19]. Jones et al. [20] investigated the cognitive abilities of 100 adolescents with ASD using ten tasks to measure the domains of ToM and executive function and found that ability in ToM was associated with both social communication symptoms and RRBs.

In the past decade, researchers have confirmed the correlation between ToM and social interaction and considered ToM a vital strategy to enhance social communication and interaction skills [4]. Apperly [21] suggested that superior theory of mind performance benefits social competence over and above any influence of general cognitive factors (e.g., language, executive function). Data are based largely on 3- to 6-year-old typically developing children points to an association between theory of mind and prosocial behavior, peer popularity and reciprocated friendship [22,23,24]. In adolescence, the social lives of children spend more time with peers outside the family [25]. The deficit in ability to build, manage and maintain social relationship in early adolescence matters lead to poorer academic outcomes and difficulties in work [26,27]. However, only several studies have focused on improving the social competence of middle children and early adolescence with ASD. Ozonoff and Miller [28] examined the effectiveness of a social skills training program that embedded social-cognitive principles for average-IQ adolescents with autism and demonstrated significant improvement in the treatment group compared to the control group. Begeer et al. [29] examined the effectiveness of ToM treatment and reported that, even though the treated adolescents with ASD improved in their conceptual ToM skills, their social communication did not improve significantly. Similarly, a study by Marraffa and Araba [30] demonstrated that children with autism did not strengthen their social interaction abilities purely due to ToM-based interventions. More recently, Lecheler et al. [31] investigated the efficacy of the Teaching ToM curriculum. The study indicated that parents noticed their children improved in social understanding post-intervention, although direct measures of ToM did not demonstrate changes. According to these results, the effectiveness of using ToM treatment itself manifested not always significant.

With the increased need of decreasing social interaction deficits observed in adolescence with autism, a combination of intervention strategies has been advocated. One study demonstrated the effectiveness of combining ToM with social skills training into one intervention package. Feng et al. [32] combined ToM components (e.g., desire-related emotion, basic beliefs and false beliefs) and social training skills (e.g., expressing emotions and communication) on an 11-year-old student with high-functioning autism and demonstrated the improvement of this novel combination of intervention strategies on social competence of student with ASD. Furthermore, Feng et al. [33] found that individuals who have perspective-taking abilities are able to look beyond their personal points of view and consider other people’s perspectives, which is a foundational ability of “praising others” that is a core social interaction skill. Moreover, distinguishing “situation-based emotion” is a prerequisite skill of a help-seeking behavior [33]. As one of the fundamental ToM skills, “desire-based emotion” refers to the understanding of causal relationships related to desires, beginning with the desire, to intentional action, to outcome, and eventually, ending with the emotional consequences of the outcome [28]. Thus, different components of ToM combine with the corresponding social interaction skills may have a better effectiveness of intervention. However, very little research has explored how ToM combined with social skills training affect social competence in adolescence with ASD.

The current study reported in this paper extended Feng et al.’s prior empirical work by using discrete-trial teaching (DTT) and conducting a multiple baseline across behaviors design on three adolescents with ASD. DTT is a commonly used procedure, particularly in early intervention settings [20]. It involves the delivery of single trials involving three core components. First, the therapist delivers the instruction. A prompt is delivered to assist the child to respond correctly. Finally, the response is reinforced [34]. By evaluating the outcomes of intervention, the researchers further investigated the effect of ToM-SS on improving students’ social communication skills. Specifically, this study tested two research questions: (1) Does ToM-SS program affect positively the improvement of social communication skills of adolescents with autism? and (2) Do improved social communication skills of adolescents with autism from ToM-SS program get generalized?

## 2. Materials and Methods

### 2.1. Participants

Three Chinese adolescents with ASD were selected from a special education school in Hangzhou. All the participants met the following eligibility criteria: (1) diagnosis of ASD based on DSM-5 by a licensed psychologist or physician with extensive experience in early identification of children with ASD; (2) Wechsler Intelligence Scale for Children (WISC-4) score ranging from 50 to 70; (3) no concurrent neurological abnormalities and no recent drug treatment; (4) normal vision and hearing abilities with basic verbal, listening and comprehension skills; (5) no recent participation in any ToM interventions; and (6) signed participant and parental informed consent before participating. Then for all participants, the Chinese version of the Social Responsiveness Scale (SRS) [35] and the Childhood Autism Rating Scale (CARS) [36], to assess levels of social impairment and autism symptoms, was completed by the parents of adolescents with ASD. Moreover, the Test of Theory-of-Mind (TToM) [32] was used for adolescents with ASD to determine the severity of their social skill deficits.

Participant 1. He was an 18-year-old student in the third year of vocational class and obtained a score of 31 on the CARS. In terms of social behavior, Participant 1 obtained a T-score of 78 on the SRS, and a total score of 3 on the TToM. He had a weak understanding of why questions, but he was able to understand simple what and who questions, continuous action instructions and basic causal relationships. He had sufficient imitative language and was able to participate in basic conversations to answer questions, while initiate questions in this adolescent with autism was deficit. Although he was able to express needs using simple sentences, his ability to express rejection had not emerged. His initiated questions were mostly meaningless and repetitive.

Participant 2. He was a 12-year-old student attending the sixth grade. The score of the CARS of this student was 32.5 and indicated that he was mildly autistic. The T-score of the SRS was 80, and the total score of the TToM was 0, which means that this student has some basic social behaviors. Specifically, he was able to understand *what* and *who* questions yet struggled to understand causality. Like Participant 1, his initiated questions were predominantly meaningless and repetitive. Although Participant 2 was able to answer basic questions using imitative language, he was unable to take the initiative to ask questions and unable to express rejection.

Participant 3. He was a 17-year-old student attending the second year at a vocational class. He obtained a score of 34 on the CARS, meaning this student was moderate autistic. His social skills were assessed, resulting in a *T*-score of 84 on the SRS, and a total score of 4 on the TToM. Specifically, he was able to understand what and who questions, sequential instructions on actions to follow and simple causal relationships. He had mastered the skills of answering questions and using complex sentence structures to express needs.

The study was approved by the Ethics Committee of Center for Cognition and Brain Disorders in Hangzhou Normal University (HR 20190605) and was in line with the guidelines of the Declaration of Helsinki. All participants and their parents gave written informed consent before the experiment.

### 2.2. Setting

The present study was conducted in a resource room in the school where ToM and social skills training via DTT (Discrete Trial Teaching) was performed in a one-to-one format. All participants were familiar with the resource room since they had visited it several times before training began. A video camera was set on a tripod in one corner of the room to record all training sessions, and this study only acquire the recordings of two children with autism because the parent of the third child with autism refused to record by the camera. The resource room had a square desk in the central with three chairs for the students. During the intervention phase, the student always sat to the right of the teacher.

### 2.3. Target Selection

Target ToM behaviors and social interaction behaviors were selected based on the participants’ results on the Test of Theory of Mind (TToM) and the results on the Social Responsiveness Scale completed by the teacher (SRS). The items of TToM score 0 and the items of SRS scores below 5 would be treated as the target ToM behaviors and the target social interaction behaviors for a participant. In the current study, the summary of the target behaviors for each participant could be found in Table 1.

### 2.4. Materials

The present study used the following three types of materials for intervention and evaluation: electronic version pictures, color-printed cards and social videos. The electronic version of the pictures came from three ways of editing the publicly available online pictures, taking real-life photos and hand-painted pictures, and then three experts in the field of special education and two teachers from special education schools were invited to evaluate the consistency of these pictures. At last, we obtained two groups of pictures for intervention. Among each group, there were seven real-life pictures, which included 3 pictures of people familiar with the participants (e.g., teachers, classmates and parents) and 4 pictures of strangers, and 3 cartoon pictures. Actually, these cartoon pictures consist of a smiley face of human and animals, and a square face of other cartoon characters. All pictures were presented through Microsoft Office PowerPoint 2010.

Next, color-printed cards were obtained by printing the above-mentioned electronic version of pictures in color printing and plastic packaging. The size of each card was 23.5 cm × 16.5 cm to ensure that the content in the card is complete and clear. Finally, “praising others” as one of social interaction target behaviors needs to be presented in a dynamic situation, thus based on the principle of video modeling and the teaching goal of “praising others”, the scene of the interaction between two actors was recorded and a social interaction video was made. A total of four social videos were used for intervention.

### 2.5. Dependent Variables and Measurements

**ToM Skills.** According to the measurement of ToM skills of Feng et al.’s study, we used the same theory of mind test (TToM). The TToM was reported to have a reliability score of 0.78 to 0.84 and a content validity score of 0.62–0.93 in a Chinese sample [18]. The TToM consist of 39 questions across eight vignettes representing situation-based emotions, desire-based emotions, basic belief, first-order false belief, second-order false belief and fact-, recall- or hint-type questions. The question in the TToM were divided into three levels. Level 1 refers to the ability to identify others’ situation-based and desire-based emotions, basic beliefs and facts related to the vignettes. It consists of 22 questions with each scoring 1 (correct) or 0 (incorrect) for a total score ranges from 0 to 22. Level 2 comprises 15 questions that two of them are scored between 0 and 2 (0-incorrect, 1-understanding that seeing leads to knowing, 2-understanding first-order belief) and the rest of them are scored either 1 (correct) or 0 (incorrect). This level represents the ability to identify first-order false belief, and the total score for Level 2 ranges from 0 to 17. Finally, Level 3 concerns the ability to identify second-order false belief and consist of only two questions, one scoring either 1 (correct) or 0 (incorrect) and another scoring between 0 and 2 (0-incorrect, 1-understanding first-order belief, 2-understanding second-order belief), and the total score for Level 3 ranges from 0 to 3. Therefore, the overall score on the TToM ranges from 0 to 42. The TToM has been used to assess the effect of ToM training for an adolescent with ASD by measuring behavioral change before and after an intervention.

The measurement of ToM skills in the current study was assessed during baseline, intervention and maintenance phases, and at the end of each training session using multimedia visual presentation (i.e., photographs displayed in the Microsoft Office PowerPoint presentation and a social video) on a laptop computer and some colored cards. Each evaluation consisted of ten items of similar situation-based scenarios related to the ToM skill being taught. Each item was preceded with a brief scenario, within which participant was instructed to perform the target skill (i.e., ToM skills 1–3). An example of the scenario and an evaluation is as follows: “Eric is staring at an opened box, do you think Eric knows what inside of the box?” All the scenarios reflected situations the participants would encounter in their daily life. The ten scenarios for each skill had the same question structure; however, none of the scenarios was repeated. All evaluation items were reviewed by three professionals, which included one special education teachers and two professors of special education in a teacher education university, to ensure the clarity and appropriateness of the items. The number of correct responses on each evaluation probe was divided by ten items on the probe and then multiplied by 100 to yield a percentage correct response for each ToM skills.

**Social Interactions.** The measurement of social interaction skills in this study used the Chinese version of the SRS. The Chinese version of SRS was developed by the Taiwan Autism Study Group which is led by Gau and Wu, with permission from Dr. Constantino and under the approval of Western Psychological Services in 2008. It consists of 65 social behavior descriptions without any judgmental overtone. The SRS is designed as a self-or caregiver-report four-point Likert-type questionnaire in regard to the frequency of each behavior (“1” never true and “4” always true) for quantifying autistic traits, and it can be divided into five subscales for intervention (i.e., social awareness, social cognition, social communication, social motivation and autistic mannerisms) [37].

Social interactions were also measured during baseline, intervention and maintenance phases, and at the end of each training session using multimedia visual presentation (i.e., photographs displayed in the Microsoft Office PowerPoint presentation and a social video) on a laptop computer and some colored cards. There were ten evaluation items related to the similar scenarios for each social interaction skill being taught, and the participant was instructed to perform the target skill (i.e., social interaction skills 1–3). An example of the scenario and an evaluation is as follows: “When Eric (one of participant’s classmates) unable to reach an object on a bookshelf, what would Eric do to ask for help?” The ten scenarios for each skill had the same question structure and none of these scenarios was repeated. All evaluation items were also reviewed by three professionals and then to calculate a percentage correct response for each social interaction skills.

### 2.6. Procedure

A multiple baseline across behaviors design was applied to evaluate the effects of the ToM-SS program on the participants’ acquisition and maintenance of those target behaviors. Primary data collection and the training of participants was carried out by a second-year graduate student of special education who had received research training and completed a one-semester practicum in a special education classroom. At the intervention phase, there was an undergraduate student of special education as the observer, who had received training and sat behind the intervention performer to simultaneously record the data.

**Pretest and Posttest.** At the beginning of the study, all three participants completed the Social Responsiveness Scale (SRS) [35], the Childhood Autism Rating Scale (CARS) [36] and the Test of Theory-of-Mind (TToM) [32] to meet the selection criteria for participation in this study and assess their social skill deficits. Then, the TToM and the SRS were used to assess the social skills of the three participants after intervention training.

**Probe Session across Conditions.** Probe sessions for each target behaviors were conducted across baseline, intervention and maintenance conditions. Each probe session comprised 10 probe trials and lasted approximately 10 min. A probe trial for each target behavior was implemented in the following steps. First, the instructor gave verbal direction—“Listen!”—to obtain a participant’s attention. Secondly, the instructor verbally described the scenario with showing a picture or a colored card, then asked questions related to the scenario and waited until the participant to respond. No matter what the participant responded and if either response was correct or not, the instructor provided praise only to reinforce the participant’s attending behavior (e.g., “You are sitting right here” or “Thank you for your listening carefully”). The probe session ended when all questions for a scenario had been asked and the participant’s responses were recorded.

**Intervention.** Each Participant was taught two sets of skills, one set included two target behaviors of the ToM and another set contained two target behaviors of the social interaction skills. For each participant, every target behavior training was introduced once a stable baseline (the number of consecutive correct response more than 2 times or a stable unimproved trend) was established. The intervention procedure of one target behavior was divided into training and probe session. The same target behavior kept the same procedures. For the training session, there were two questions related to one target behavior would be repeated three times. The instructor verbally described the scenario with showing a picture or a colored card, then asked questions related to the scenario and waited until the participant to respond. If the participant did not respond or responded incorrectly, the instructor would provide the oral or gesture prompts to help participant say the correct answer, and then ask the participant to imitate the instructor to say the correct answer. If the participant responded correctly, the instructor would provide agreement sentences (e.g., “Yes, you feel sad.”) and continued. Training continued until the participant’s performance reached the criterion (above 90% accuracy for one target behavior). After that the probe session would repeat two questions once again. For example, the intervention procedures of expressing emotion that was one of social interaction target behaviors shown in the Table 2. The duration of intervention lasted for 15 weeks, from September to December 2019. Training (at least 35 min each time) and probe session was conducted four times a week, 5 min interval between training and probe session to allow the participants to rest. A target behavior would enter the maintenance phase after one week of completion of intervention. In order to understand the maintenance effect of those target behaviors in three participants, the experimenter did not give any teaching or prompts to the participants and collected data by the probe program. The maintenance phase lasted three days.

We compiled the Teaching Record Sheet (TRS) to record the data. The TRS includes the probing procedure record sheet and intervention procedure checklist for the baseline, intervention and maintenance phases. The probing procedure record sheet documents the percentage of learning changes in the target behavior; the intervention procedure checklist is used as an implementation fidelity check.

**Reliability and Fidelity.** To ensure scoring reliability, approximately 20% of the intervention tapes were randomly selected and scored by two researchers. Observation agreement was reached when both researchers recorded an occurrence or a nonoccurrence in the same interval for each data probe. Interobserver agreement was then calculated for each variable by dividing the number of agreements by the total number of agreements plus disagreements and multiplying the result by 100. The mean occurrence agreement for the learning outcome evaluation probes was 96.8% for Participant 1, 97.5% for Participant 2 and 95.5% for Participant 3.

Procedural integrity was monitored through a training fidelity checklist created by the researcher. Thirty percent of the intervention tapes were observed and scored. The intervention sessions were implemented with 100% procedural fidelity during the selected probes.

**Social Validity.** The current study developed the questionnaire and interview script with reference to Long’s [38] social validity survey. The teacher version of the questionnaire contained eight questions that included a satisfaction survey and open-ended questions. The parent version of the questionnaire included 17 questions that covered topics related to acceptability, convenience and satisfaction, as well as open-ended questions. The student version interview script included four questions that mainly focused on learning satisfaction. The teacher and the main caregiver completed the paper-based questionnaires, and the student participants answered the four questions through interviews.

## 3. Results

### 3.1. The Training Performance of Each Participant

**Figure 1:**
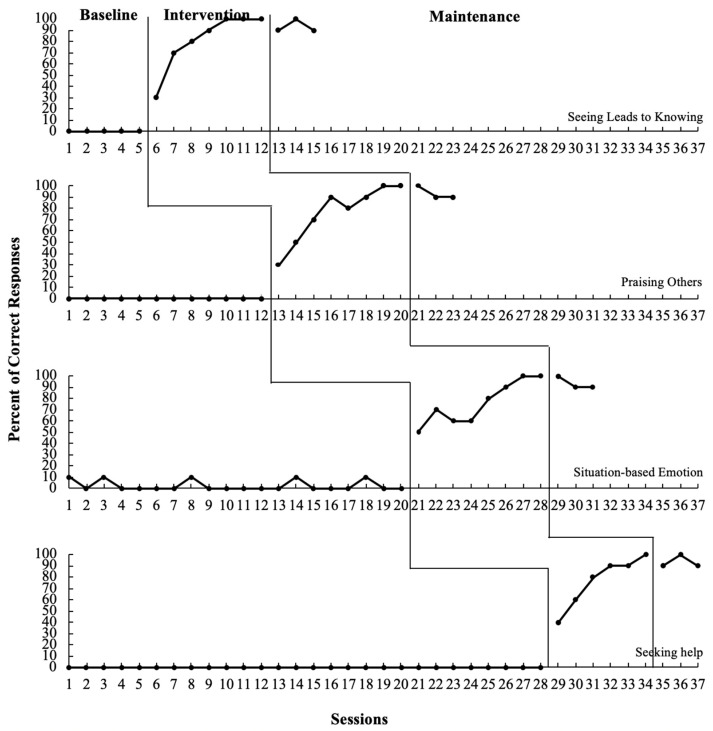
Percentage of correct responses from Participant 1.

Participant 1. Visual analysis revealed an immediate and obvious change in level and an upward trend after introducing the ToM-SS (refer to Table 3 and Figure 1). Participant 1′s target behaviors—seeing leads to knowing, praising others, identifying situation-based emotion and seeking help—were successfully generalized and maintained after the treatment condition.

**Figure 2:**
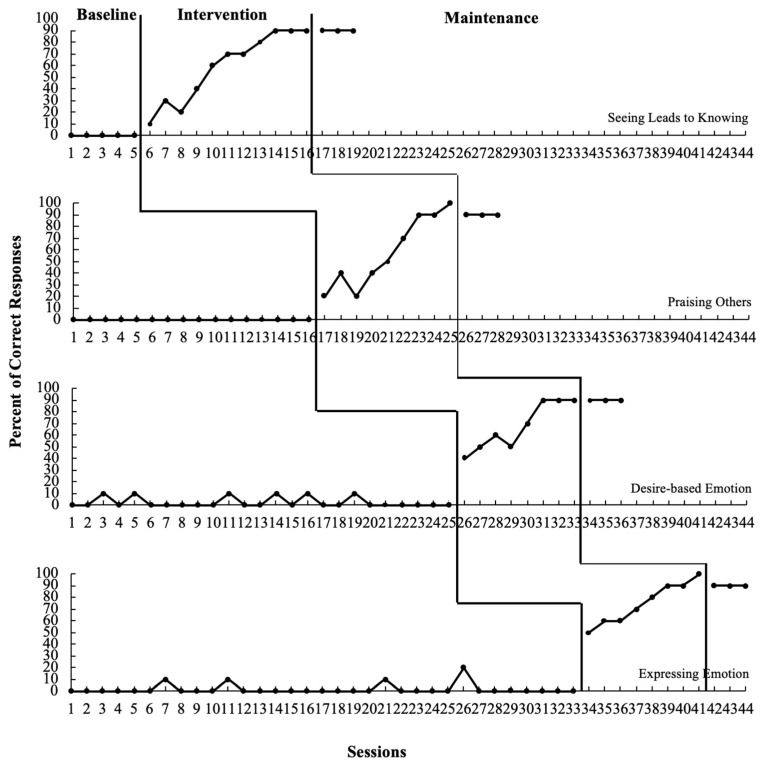
Percentage of correct responses from Participant 2.

Participant 2. Visual analysis revealed an immediate and obvious change in level and an upward trend after introducing the intervention (refer to Table 4 and Figure 2). The data support the effectiveness of the ToM-SS in teaching social skills to Participant 2, and most of the newly learned skills were effectively maintained after the treatment condition.

**Figure 3:**
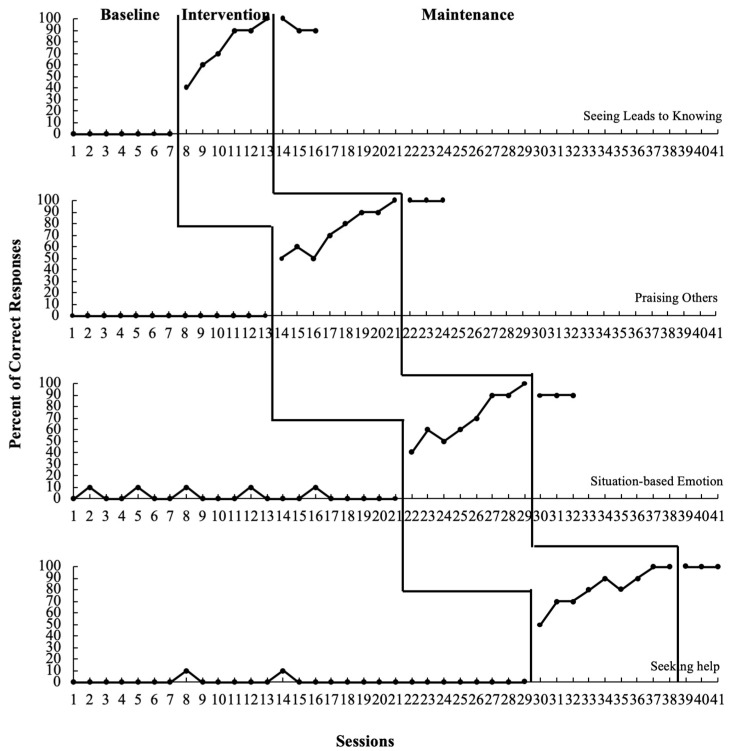
Percentage of correct responses from Participant 3.

Participant 3. The introduction of the intervention resulted in an increase in the percentage of correct responses. The graph (refer to Table 5 and Figure 3) shows a steep, increasing trend with high stability once the intervention commenced. Participant 3′s target behaviors—seeing leads to knowing, identifying situation-based emotion, seeking help and praising others—were successfully generalized and maintained after the treatment condition.

### 3.2. Pretest and Posttest Scores on the TToM

Pretest and posttest scores on the TToMs of the three participants are presented in Table 6. For the total score, Participant 1 scored 3 on the pretest and 10 on the posttest; his scores on Level 1 were 3 and 8 on the pretest and posttest, respectively; he scored 0 on the pretest and 2 on the posttest at Level 2. For the total score, Participant 2 scored 0 on the pretest and 8 on the posttest; his scores on Level 1 were 0 and 6 on the pretest and posttest, respectively; he scored 0 on the pretest and 2 on the posttest at Level 2. For the total score, Participant 3 scored 4 on the pretest and 10 on the posttest; his scores on Level 1 were 4 and 8 on the pretest and posttest, respectively; he scored 0 on the pretest and 2 on the posttest at Level 2. All three participants demonstrated an increase in ToM, and especially made progress in beginner and intermediate level skills.

### 3.3. Pretest and Posttest Scores on Social Skills

Pretest and posttest scores on the social skills of the three participants are shown in Table 7. Score reductions in the SRS are associated with a decrease in observable social skills related to ASD symptoms. After Participant 1 received the training, his T-score decreased from 78 to 71, which included a decrease in the communication score from 18 to 13 and a decrease in the motivation score from 12 to 10. Reflected by the SRS assessment, his major improvements were in three areas: being aware of what others are thinking or feeling, being able to communicate his feelings to others, and starting social interactions with peers or adults.

After Participant 2 received the training, his *T*-score decreased from 80 to 73, which included a decrease in the cognition score from 14 to 12 and a decrease in the communication score from 20 to 15. According to the SRS assessment, his major improvements were in four areas: able to elicit the real meaning of a conversation, able to communicate his feelings to others, more patient trying to get ideas across in conversation, and less socially awkward when trying to be polite to others.

Participant 3′s *T*-score decreased from 84 to 78 after receiving the intervention; this included a decreased in the communication score from 22 to 18 and a decrease in the motivation score from 12 to 10. The SRS assessment indicates his major improvements were in three areas: able to communicate his feelings to others, more patient trying to get ideas across in conversation, and less difficulty relating to adults.

Although the three participants’ SRS scores were still above 70 after the intervention, their scores in the fields of social cognition, social communication and social motivation decreased by varying degrees, indicating that all participants’ social communication skills improved to a certain extent.

### 3.4. Social Validity

Social validity was assessed through surveys and interviews with students, teachers and primary caregivers. The findings indicated that all educators agreed that learning these target behaviors was a developmentally appropriate goal for the participants. The results also suggested that the teachers’ average satisfaction score regarding the teaching plan was 4.4 (*SD* = 0.49). The acceptance rate from all caregivers pertaining to the teaching plan was 5 (*SD* = 0), the average satisfaction score was 4.4 (*SD* = 0.49), and the average convenience score was 5 (*SD* = 0). The results illustrated that teachers, caregivers and students were all highly satisfied with and accepting of the teaching plan.

In the open-ended questions, Participant 1′s teacher reported that Participant 1 had made major improvements in his ability to understand other people’s emotions. For example, when seeing his teacher is upset, Participant 1 says, “The teacher is angry; we should be quiet.” In a real-life context, even though Participant 1 is unable to proactively praise others, in situations similar to those in the intervention program, he can praise others with the teacher’s prompts. Participant 2′s primary caregiver reported that his ability to express his emotions has improved. Before participating in the intervention program, he frequently felt anxious about environmental changes and jumped around in the classroom. He can now sit quietly in place under the teacher’s guidance and express emotions such as, “I feel sad.” Participant 3′s teacher mentioned that he improved his ability to pay attention to peers and became more insightful about others’ feelings after the training. When seeing that a classmate seems upset, he will say, “She is angry.” He is also able to make positive comments to others when noticing that they are wearing new clothes.

## 4. Discussion

This study investigated the effects of the ToM-SS training program on improving ToM and social interaction skills of three adolescents with ASD. The percentage of correct responses indicated remarkable improvement in participants’ performances after implementing the ToM-SS training. Data analysis revealed a functional relationship between intervention and accuracy of responses.

In the areas of ToM, the current study found that Participants 1 and 3 made substantial improvements in their ToM target behaviors. These results may be related to their relatively sufficient cognitive and language abilities. In contrast, Participant 2 had the weakest cognitive and language abilities, which may affect the intervention program’s effectiveness in improving his skills. This finding is consistent with previous studies’ conclusions that interventions rooted in applied behavior analysis can promote ToM acquisition [39]. This finding is also aligned with Zhang’s [40] finding that the thought bubbles (representing what the person is thinking) can improve ToM abilities in children with low-functioning autism. Compared with Long’s [38] study that adopted multiple examples of teaching methods, although the target behaviors were different from those in the current study, both studies adopted a multiple baseline across behaviors design and demonstrated that ToM skills can be improved through structured interventions. While previous studies focused solely on improving ToM ability, this study primarily aimed to enhance the participants’ performances by combining ToM and social skills training into one intervention package.

Furthermore, data on the TToM scores show that all participants’ TToM scores improved significantly following the intervention. Participant 2 made more significant progress—his total scores increased from zero to eight. His pretest score of desire-related emotions was zero, and his posttest scores for the same measure was four. He also experienced a collateral gain because his posttest scores on this measure also improved despite no direct intervention to improve his basic belief. A similar situation was manifested to varying degrees in the other two students. These results may indicate that the structural training of a ToM specific behavior might facilitate other ToM capabilities. However, it is worth noting that improved ToM was limited to basic level. The scores for advanced ToM questions did not yield much change, suggesting that the acquisition of advanced ToM requires the students to fully master basic and intermediate abilities and have a considerable degree of cognitive and communication skills.

The results of social validity indicated that all participants’ ToM had improved to varying degrees in their daily lives. However, Lecheler et al. [31] reported no significant changes in young children with ASD. The age of the participants in the current study and Lecheler et al.’s study differed. This may have led to different results. In our study, the participants were adolescents. In fact, our results are aligned with the researchers’ expectations and further validates the results of Feng et al. Their research concluded that ToM interventions combined with social skills training can significantly improve students’ ToM ability [32].

In this study, the positive results in the social interaction scores on all the participants can be attributed to individuals’ abilities. For example, Participant 3 made the improvements in target social behaviors. This result may be related to the fact that he had stronger social motivation and higher frequency of active social behaviors before participating in the intervention. Consistent with previous studies’ conclusions, interventions combined with video modeling can effectively improve the social skills of children with autism [41]. The current study also verified previous studies that reported that social skills training can effectively improve the social communication and interaction skills of individuals with ASD [42,43]. In addition, all participants’ social communication skills significantly improved after the intervention, which was reflected in SRS as the decrease in *T*-score and subscales of cognition, communication and motivation. Among them, Participant 3 made the best progress. His *T*-score decreased from 84 to 78, which may be related to his higher cognitive ability and stronger social motivation. Participant 1′s target skills—praising others and seeking help—were selected from SRS’s communication subscale. His motivation subscale scores also improved post-intervention, indicating that stronger social communication skills may increase social motivation. Similarly, the social validity survey indicated that the participants’ social communication skills improved to varying degrees in their daily lives. The combination of ToM and social skills training can effectively improve the participants’ social communication skills and maintenance, consistent with previous research [32]. However, the present study was unable to collect generalization data and only used the changes in social scores and ToM scores to analyze students’ improvements in abilities. In addition to using digital pictures and graphic cards, the video modeling technique was adopted to teach “praising others” and strengthen the external validity among three participants.

Before closing the discussion, several limitations of this study need to be addressed in future research. First, a lack of social skills generalization data required the researchers to limit the scope of the follow-up analysis. Due to practical constraints, the researchers used the teacher-completed SRS pre- and postintervention scores to measure students’ social performance. This approach was inevitably affected by teachers’ subjective judgments. Future research is needed to conduct systematic data collection on generalization measures and evaluate generalization effects with rigorous experimental control. Future studies should also consider adding observations in natural settings and collecting data points through video recordings, which may maximize generality effects while examining the functional relationship between intervention and corresponding social skills.

Another limitation involved the presentation format of the teaching materials, which was mainly cards, slides and videos. Although these formats were considered easy to follow, they still lacked variability and could have included more engaging features. Future studies may consider modifying the training materials to appropriately reflect natural settings and students’ learning experiences. For nonverbal participants, the study can be designed to incorporate personal work system principles to improve the operability of teaching materials.

Last but not least, ToM-SS was solely conducted by researchers in structured classroom settings. Future research is required to determine the extent to which benefits can be achieved with teachers and nonprofessionals (e.g., parents and peers). More research is needed, especially to explore the hypothesis that parent- or peer-directed formats and less-controlled settings could have long-term effects on social skills generalization. Finally, there was a small sample size (N = 3) in our study. In order to maximize generality effects, future studies need to implement social skills interventions with a larger number of students with ASD.

## 5. Conclusions

This study which sought to explore the effect of ToM-SS program on the social competence of students with ASD yielded some preliminary promising results. After receiving the ToM-SS program training, all three participants significantly improved their ToM skills and other social skills. Feedback and comments from teachers and parents also suggest that some of these ToM and social skills have been generalized to their daily routines which indicates good social validity of the study.

## Figures and Tables

**Table 1:** Target behaviors and operational definitions.

Participant	Domain	Target Behavior	Operational Definition	Example
1, 2, 3	ToM1	Seeing leads to knowing	Able to judge whether a person knows the content of a covered object by observing the person’s looking direction.	When Eric is staring at a box, the student would be able to judge whether Eric knows what is inside the box.
1, 2, 3	Social Skill1	Praising others	Able to verbally compliment others when seeing them do something positive.	When seeing Eric complete a long-distance basketball shot, the student would say, “Great job!”
1, 3	ToM2	Situation-based emotion	Able to tell others’ emotions (e.g., happy, sad) based on situational contexts.	When seeing Eric’s toy is broken, the student would understand how Eric feels.
1, 3	Social Skill2	Seeking help	Able to verbally ask for help when encountering difficulties.	When unable to reach an object on a bookshelf, the student would verbally ask an adult for help.
2	ToM3	Desire-based emotion	Able to tell others’ emotions (e.g., happy, sad) based on their emotional desire.	When Eric finally received the toy he always wanted, the student would be able to tell that Eric feels happy.
2	Social Skill3	Expressing emotion	Able to verbally express emotions.	When the student feels sad, they would be able to verbally express the emotion.

**Table 2:** An example of the intervention procedure: expressing emotion.

	Intervention Instruction	Student Response	Teacher Feedback
Training	Present pictures and ask questions:		
	Q1: Suppose you are this kid, and you dropped your ice cream cone. How would you feel?	(1) The correct answer: I would feel very sad (student needs to use “I” in the sentence structure). (2) No response or wrong answers.	(1) For the correct answer: Yes, you feel sad. (descriptive reinforcement) (2) For no response or wrong answers: - Oral/gesture prompts for answers. - Imitate and say the correct answer.
	Q2: Why do you feel sad?	(1) The correct answer: Because I dropped my ice cream cone. (2) No response or wrong answers.	(1) For the correct answer: Yes, you dropped your ice cream cone, so you feel sad. (descriptive reinforcement) (2) For no response or wrong answers: - Oral/gesture prompts for answers. - Imitate and say the correct answer.
Probing	Present pictures and ask questions:		
	Q1: Suppose you are this kid, and you dropped your ice cream cone. How would you feel?	(1) The correct answer: I would feel very sad (student needs to use “I” in the sentence structure). (2) No response or wrong answers.	Yes, you listen to me carefully. (attitude reinforcement)
	Q2: Why do you feel sad?	(1) The correct answer: Because I dropped my ice cream cone. (2) No response or wrong answers.	Good! You are sitting here quietly. (attitude reinforcement)

**Table 3:** Visual analysis for the target behaviors of Participant 1.

In-phase analysis:												
Target Behavior	ToM1			SS1			ToM2			SS2		
Sequence	A	B	C	A	B	C	A	B	C	A	B	C
Length	5	7	3	12	8	3	20	8	3	28	6	3
Trend	—	/	—	—	/	\	—	/	\	—	/	—
Trend Stability	100%	75.4%	100%	100%	75%	100%	100%	75%	100%	100%	75%	100%
Average	0	81.43	93.33	0	76.25	93.33	2.5	76.25	93.33	0	76.67	93.33
Level Range	0–0	30–100	90–100	0–0	30–100	90–100	0–10	50–100	90–100	0–0	40–100	90–100
Level Stability	100% S	28.6% US	100% S	100% S	25% US	100% S	100% S	25% US	100% S	100% S	16.7% US	100% S
Level Change	0–0 (=)	30–100 (+70)	90–90 (=)	0–0 (=)	30–100 (+70)	100–90 (−10)	10–0 (−10)	50–100 (+50)	100–90 (−10)	0–0 (=)	40–100 (+60)	90–90 (=)
C Value	—	0.76	−0.5	—	0.83	0.25	−0.20	0.79	0.25	—	0.80	−0.5
Z Value	—	2.34 **	−1.41	—	2.69 **	0.71	−0.94	2.55 **	0.71	—	2.37 **	−1.41
**Between-stages analysis:**												
Target Behavior	ToM1			SS1			ToM2			SS2		
Comparison	A/B		B/C	A/B		B/C	A/B		B/C	A/B		B/C
Trend Change	/ (+)		— (=)	/ (+)		\ (−)	/ (+)		\ (−)	/ (+)		— (=)
Change Between Levels	0–30 (+30)		100–90 (−10)	0–30 (+30)		100–100 (=)	0–50 (+50)		100–100 (=)	0–40 (+40)		100–90 (−10)
Percentage Overlap	0%		100%	0%		100%	0%		100%	0%		100%
C Value	0.94		0.38	0.96		0.44	0.93		0.25	0.96		0.46
Z Value	3.55 **		1.12	4.53 **		1.25	5.13 **		0.98	5.76 **		1.43

Note: ** *p* < 0.01; S = Stable; US = Unstable.

**Table 4:** Visual analysis for the target behaviors of Participant 2.

In-phase analysis:												
Target Behavior	ToM1			SS1			ToM2			SS2		
Sequence	A	B	C	A	B	C	A	B	C	A	B	C
Length	5	11	3	16	9	3	25	8	3	33	8	3
Trend	—	/	—	—	/	—	—	/	—	—	/	—
Trend Stability	100%	81.8%	100%	100%	77.8%	100%	100%	75%	100%	100%	75%	100%
Average	0	59.1	90	0	57.8	90	2.4	67.5	90	1.5	75	90
Level Range	0–0	10–90	90–90	0–0	20–100	90–90	0–10	40–90	90–90	0–20	50–100	90–90
Level Stability	100% S	9.1% US	100% S	100% S	0% US	100% S	100% S	12.5% US	100% S	100% S	25% US	100% S
Level Change	0–0 (=)	10–90 (+80)	90–90 (=)	0–0 (=)	20–100 (+80)	90–90 (=)	0–0 (=)	40–90 (+50)	90–90 (=)	0–0 (=)	50–100 (+50)	90–90 (=)
C Value	—	0.91	—	—	0.85	—	−0.32	0.81	—	−0.12	0.89	—
Z Value	—	3.32 **	—	—	2.89 **	—	−1.64	2.64 **	—	−0.72	2.87 **	—
**Between-stages analysis:**												
Target Behavior	ToM1			SS1			ToM2			SS2		
Comparison	A/B		B/C	A/B		B/C	A/B		B/C	A/B		B/C
Trend Change	/ (+)		— (=)	/ (+)		\ (−)	/ (+)		\ (=)	/ (+)		\ (−)
Change Between Levels	0–10 (+10)		90–90 (=)	0–20 (+20)		100–90 (−10)	0–40 (+40)		90–90 (=)	0–50 (+50)		100–90 (−10)
Percentage Overlap	0%		100%	0%		100%	0%		100%	0%		100%
C Value	0.96		0.24	0.95		0.44	0.93		0.38	0.94		0.21
Z Value	4.09 **		0.86	4.96 **		1.73	5.53 **		1.12	6.18 **		0.83

Note: ** *p* < 0.01; S = Stable; US = Unstable.

**Table 5:** Visual analysis for the target behaviors of Participant 3.

In-phase analysis:												
Target Behavior	ToM1			SS1			ToM2			SS2		
Sequence	A	B	C	A	B	C	A	B	C	A	B	C
Length	7	6	3	13	8	3	21	8	3	29	9	3
Trend	—	/	\	—	/	—	—	/	—	—	/	—
Trend Stability	100%	75%	100%	100%	87.5%	100%	100%	87.5%	100%	100%	77.8%	100%
Average	0	75	93.33	0	74	100	2.4	70	90	0.7	81.1	100
Level Range	0–0	40–100	90–100	0–0	50–100	100–100	0–10	40–100	90–90	0–10	50–100	100–100
Level Stability	100% S	16.7% US	100% S	100% S	25% US	100% S	0% US	12.5% US	100% S	93.1% S	22.2% US	100% S
Level Change	0–0 (=)	40–100 (+80)	100–90 (=)	0–0 (=)	50–100 (+80)	100–100 (=)	0–0 (=)	40–100 (+50)	90–90 (=)	0–0 (=)	50–100 (+50)	100–100 (=)
C Value	—	0.80	0.25	—	0.83	—	−0.31	0.81	—	−0.07	0.78	—
Z Value	—	2.38 **	0.71	—	2.68 **	—	−1.50	2.63 **	—	−0.41	2.65 **	—
**Between-stages analysis:**												
Target Behavior	ToM1			SS1			ToM2			SS2		
Comparison	A/B		B/C	A/B		B/C	A/B		B/C	A/B		B/C
Trend Change	/ (+)		\ (−)	/ (+)		— (=)	/ (+)		\ (−)	/ (+)		— (=)
Change Between Levels	0–40 (+40)		100–100 (=)	0–50 (+50)		100–100 (=)	0–40 (+40)		100–90 (−10)	0–50 (+50)		100–100 (=)
Percentage Overlap	0%		100%	0%		100%	0%		100%	0%		100%
C Value	0.94		0.23	0.94		0.37	0.84		0.20	0.96		0.21
Z Value	3.66 **		0.86	4.54 **		1.11	3.07 **		0.62	6.07 **		0.78

Note: ** *p* < 0.01; S = Stable; US = Unstable.

**Table 6:** Changes in TToM scores.

Participant	Stage	Scores			Total
		Basic	Intermediate	Advanced	
1	Pretest	3	0	0	3
2		0	0	0	0
3		4	0	0	4
1	Posttest	8	2	0	10
2		6	2	0	8
3		8	2	0	10

**Table 7:** Changes in SRS scores.

Participant	Stage	Scores					Total
		Perception	Cognition	Communication	Motivation	Behavior	
1	Pretest	10	12	18	12	26	78
2		13	14	20	14	19	80
3		15	18	22	12	7	84
1	Posttest	10	12	13	10	26	71
2		13	12	15	14	19	73
3		15	18	18	10	17	78

## Data Availability

Data available on request due to restrictions e.g., privacy or ethical.
